# Uptake and Inhibition of P-Glycoprotein-Mediated Efflux Evaluation of Encapsulated Methotrexate Chitosan and Hypromellose Phthalate Nanoparticles for Potential Glioblastoma Treatment

**DOI:** 10.3390/pharmaceutics17020239

**Published:** 2025-02-12

**Authors:** Valéria de Moura Leite Naves, Rafaela Franco Dias Bruzadelli, Marisa Ionta, Maria Palmira Daflon Gremião, Liliane Neves Pedreiro, Gislaine Ribeiro Pereira, Flávia Chiva Carvalho

**Affiliations:** 1 Department of Food and Drugs, Federal University of Alfenas, UNIFAL-MG, Alfenas 37130-001, MG, Brazil; valnaves@live.com (V.d.M.L.N.); rafaela.bruzadelli@sou.unifal-mg.edu.br (R.F.D.B.); gislaine.pereira@unifal-mg.edu.br (G.R.P.); 2 Institute of Biomedicals Sicences, Federal University of Alfenas, UNIFAL-MG, Alfenas 37130-001, MG, Brazil; marisa.ionta@unifal-mg.edu.br; 3 Department of Drugs and Medicines, School of Pharmaceutical Sciences, State University of São Paulo State, UNESP, Araraquara 14800-903, SP, Brazil; pgremiao@fcfar.unesp.br (M.P.D.G.); liliane_np@yahoo.com.br (L.N.P.)

**Keywords:** chitosan, hypromellose phthalate, methotrexate, cross-linked polyelectrolyte complexes, p-glycoprotein-mediated efflux

## Abstract

**Background:** Methotrexate (MTX), a folic acid antagonist used in chemotherapy, faces limitations due to cancer cell resistance, high toxicity, and low bioavailability. **Objective:** This study developed nanoparticles (NPs) of chitosan (QS) and hydroxypropylmethylcellulose phthalate (HPMCP) to encapsulate MTX for potential effect investigation on glioblastoma cell targeting and P-gp efflux inhibition. **Method:** NPs were produced by the polyelectrolyte complexation method and were characterized by DLS, PDI, DSC, FTIR, PXRD, MEV, drug release profile, and an in vitro mucoadhesion test. Cell viability, flow cytometry, and LSCM using U251MG (glioblastoma) and CCD 1059Sk (fibroblasts) cells were used to evaluate glioblastoma and the P-gp efflux effect. **Results:** NPPM29 (QS3:1) showed 91.72% encapsulation efficiency, a mean diameter of 452.6 nm, and a zeta potential of +22.5 mV. DSC, FTIR, and PXRD confirmed the QS-HPMCP supramolecular interaction. Liquid falling mucoadhesion tests demonstrated strong retention of NPPM29 (84%) compared to free MTX (10.5%). In vitro release studies indicated controlled drug release at pH 7.4. Cytotoxicity assays in U251MG revealed enhanced efficacy of NPPM29 *(IC*_50_ = 68.79 µg/mL) compared to free MTX (*IC*_50_ = 80.54 µg/mL), with minimal impact on fibroblasts, confirming tumor specificity. Flow cytometry and LSCM confirmed improved cellular internalization and P-gp inhibition. **Conclusions:** These findings highlight the potential of MTX-QS-HPMCP-NPs for glioblastoma therapy.

## 1. Introduction

Methotrexate (MTX) is widely used as a chemotherapeutic agent in cancer treatment [1]. As a folic acid antagonist essential for DNA synthesis, MTX exhibits therapeutic effects against various cancer types due to its high specificity for tumor cells, which overexpress folate receptors on their surface. However, MTX faces significant limitations, including high toxicity and low bioavailability, since its intestinal absorption is saturable and much of the absorbed drug is rapidly metabolized by the liver, resulting in low concentrations at target tissues [2,3,4]. Moreover, clinical efficacy is compromised by cancer cell resistance, mainly due to cellular efflux and difficulties in crossing the blood–brain barrier in treating brain tumors [5].

Research has focused on developing chitosan (QS)-based nanoparticles (NPs) to target MTX delivery to tumors, especially using modified QS forms [6]. Studies have emphasized the physicochemical characterization, enhanced drug encapsulation efficiency, controlled release, drug transport, and efflux in cancer cells [7,8,9,10]. Nanoparticle systems can protect the drug from efflux, retaining higher intracellular concentrations and overcoming challenges associated with conventional formulations [6]. Nanotechnology offers significant advantages in drug development, enabling modification of physicochemical properties, improving solubility, and increasing stability. Due to their small size, nanomedicines can interact with biomolecules both on the cell surface and intracellularly, delivering drugs to action sites in ways unimaginable 40 years ago [11].

Chitosan’s amino groups form complexes with anionic molecules, and this phenomenon can be utilized for particle formation via polyelectrolyte complexation, a widely used technique for incorporating hydrophilic drugs due to its simplicity and avoidance of organic solvents and high temperatures [12]. Many researchers have explored the pharmaceutical potential of tripolyphosphate (TPP)-chitosan complexes [13,14,15,16]. However, NPs prepared by ionotropic gelation methods often suffer from aggregation, particle fusion immediately after preparation, or limited physicochemical stability during storage [12,17]. Therefore, the optimal NP formation conditions evaluation, aiming for homogeneity in size distribution and stability, is a fundamental step for producing NP using the ionotropic gelation method [12].

Hydroxypropylmethylcellulose phthalate (HPMCP) is a high-molecular-weight polymer with negatively charged carboxylic groups that can promote stronger electrostatic interactions with QS than TPP. This polymer is widely used for enhancing drug permeability [18].

Multidrug resistance is considered the most important limiting factor in cancer chemotherapy; therefore, inhibiting P-glycoprotein-mediated (P-gp) efflux and inhibiting agent development could be of great interest in cancer drug delivery systems research. Many agents have been developed to reverse the anti-cancer drug resistance, but delivering them to cancer sites and cells is difficult. The emerging nano-based drug delivery systems have been more effective in overcoming P-gp drug resistance by increasing the intracellular delivery of these agents [19]. Among NPs, biopolymeric NPs like chitosan are mainly used for cancer therapy goals because of their substantial advantages, such as cost-effectiveness, ease of manipulation, biocompatibility, mucoadhesion, biodegradability, enhanced permeation, and eco-friendly properties [20]. Although chitosan NPs have been reported in many studies related to cancer therapy, their effect on P-gp action has not been reported. Therefore, this study aims to fill the gap in this field, testing MTX-chitosan-hypromellose NPs internalization in human malignant glioblastoma multiforme cell lines (U251MG) as proof of concept.

In addition to that, these systems have significant potential for mucosal delivery (oral, buccal, rectal, vaginal, and nasal routes), as smaller particles can diffuse passively through mucus and rapidly cleared by mucociliary action. Consequently, nanotechnology has played a key role in improving mucoadhesion and has been a dominant strategy in enhancing drug release via these routes [21,22].

Given the need for an efficient drug delivery system in cancer therapy and the extensive use of QS-based NPs as nanocarriers, this work aims to develop and characterize QS and HPMCP NPs through polyelectrolyte crosslinking for MTX encapsulation, with potential applications in glioblastoma treatment. The findings of this work showed not only a greater internalization of the NPs U251MG than fibroblast normal cells (CCD 1059Sk) but also an innovative effect of the hypromellose component, showing higher U251MG cytotoxicity of NPs composed of hypromellose and chitosan than the one composed of tripolyphosphate and chitosan. This study pioneers new possibilities for functional excipients, showcasing the potential of chitosan-based nanoparticles as groundbreaking vehicles in cancer therapy. Beyond their versatility, they emerge as powerful allies in overcoming multidrug resistance, setting the stage for transformative advancements in cancer treatment.

## 2. Materials and Methods

### 2.1. Materials

Low-molecular-weight chitosan, sodium tripolyphosphate 85%, and mucin-type II were purchased from Sigma Aldrich (St. Louis, MO, USA), hypromellose phthalate HP-55 Shin-Etsu was from Chemical Co., Ltd., (Tokyo, Japan), and the methotrexate was from Fagron (Fermion, Finland). All the other materials used were of analytical grade and obtained from commercial suppliers.

### 2.2. Method of Polyelectrolyte Complexation of Chitosan

Nanoparticles were produced by low-viscosity chitosan dispersion at 4 mg.mL^−1^ in 0.1 M acetic acid, pH = 5.5, and hypromellose phthalate (HPMCP) as an ionic cross-linker at 2 mg.mL^−1^ in NaOH 1 M (pH = 5.5). Both dispersions were prepared under magnetic stirring for 30 min.

The crosslink reaction was performed by mixing HPMPC dispersion dropwise with the chitosan dispersion under magnetic stirring. Different volumes of HPMCP and chitosan solutions were tested to obtain a final volume of 4.4 mL of solutions with the required concentrations of both components. The proportions and final polymer concentrations tested are described in Table 1.

Loaded nanoparticles were prepared by dissolving MTX in phosphate buffer at 0.5 mg.mL^−1^, pH = 7.4, and incorporated into chitosan dispersion prior to the crosslink. The concentrations tested were 5% related to the total polymer weight.

Nanoparticles produced using TPP as an ionic cross-linker were replaced by HP dispersion by TPP dispersion at 2 mg.mL^−1^ *w*/*v* in water (pH = 5.5).

### 2.3. Particle Size, Zeta Potential, and Morphology

The samples were characterized by dynamic light scattering (DLS), polydispersity index (PDI), and electrophoretic mobility using a Zetasizer Nanoseries, Malvern Instruments, Worcestershire, UK. The refraction indexes used were 1.33 and 1.55 for water and chitosan, respectively. All the measurements were performed at 25 °C in triplicate.

Morphology was observed by scanning electron microscope (SEM), Leo Evo 40 XVP (Carl Zeiss), Oberkochen, Germany. Samples were prepared using nanoparticle suspensions, placed on an aluminum stub covered with a carbon adhesive disk, and coated with gold.

### 2.4. Analytical Characterization

A high-performance liquid chromatography (HPLC) analytical methodology was developed and validated to measure drug concentration. Briefly, a Shimadzu HPLC system was used for the analyses, consisting of a CBM-20A solvent delivery module, SPD-20A spectrophotometric detector (set at 306 nm), and a SIL-20AHT automatic injection module. A reverse-phase C18 column (Acqua, Phenomenex, CA, USA) of 150 mm, 4.6 mm I.D., and 5 µm particle size was used. The isocratic mobile phase was a mixture of phosphate buffer 0.05 M, pH 4.5, methanol/acetonitrile, at 83:10:7 (*v*/*v*), flowing at 1.0 mL.min^−1^, with an injection volume of 50 μL. Standard solutions of MTX dissolved in a mobile phase were prepared in triplicate with the MTX concentration ranging from 0.5 µg.mL^−1^ to 35 µg.mL^−1^. The regression line obtained was y = 158,148.1x − 13,652.1, with r^2^ = 0.999, with limits of detection and quantification of 0.011 µg.mL^−1^ and 0.038 µg.mL^−1^, respectively, and precision and accuracy within the acceptable range of ICH QR2.

### 2.5. Determination of Methotrexate Loading Efficiency

The loading efficiency was obtained by determining the concentration of MTX in the supernatant using a previously validated HPLC analytical method. The amount of MTX loaded in the nanoparticles was calculated as the difference between the total drug added and the MTX quantified in the supernatant. Loading efficiency (*LE*%) was calculated using the following equation:

(1) $$LE\%=\frac{Total\;amount\;of\;MTX-MTX\;in\;supernatant\;}{Total\;amount\;of\;MTX}\times 100$$

### 2.6. Fourier Transform Infrared Spectroscopy (FTIR)

Particles used in the analysis were previously lyophilized to produce fine powder. Then, the powder was mixed with KBr and pressed into a pellet. The FTIR spectra were recorded using an FTIR—Fourier transform infrared spectrometer Shimadzu^®^, Affinity-1 (Tokyo, Japan) coupled to a Pike Miracle^®^ attenuated total reflectance sampling accessory with ZnSe Pike Technologies^®^ crystals (Madison, WI, USA). FTIR spectra of pure polymers and MTX were also recorded for differential comparison.

### 2.7. Powder X-Ray Diffraction (PXRD) Analysis 

Crystallinity was assessed using an Ultima IV diffractometer (Rigaku, Tokyo, Japan) X-ray diffractometer from 3° to 50° (2θ) at a step of 0.05°, using Cu Kα radiation (λ = 1.5406 Å).

### 2.8. Thermal Analysis Test

About 10 mg of each dry sample was placed in an uncoated aluminum crucible under a nitrogen atmosphere and heating rates of 20 °C.min^−1^. Differential scanning calorimetry (DSC) measurements were carried out in a DSC-1 (Mettler Toledo, Gieben, Switzerland) using aluminum pans with lids. About 3.5 mg of samples were used for all the experiments, under a dynamic nitrogen atmosphere (50 mL.min^−1^), at a heating rate of 10 °C.min^−1^, and a temperature range of 25 to 200 °C in a room temperature range at 1100 °C. The crucibles used in all DSC measurements were hermetically sealed. The temperature and heat flow of the DSC instrument were calibrated with indium and zinc. A sapphire disk was used to calculate the calorific capacity.

A nano-differential scanning calorimetry (nanoDSC) instrument (TA Instruments, New Castle, UK) was also performed. Deionized water was used as white, which was discarded from all curves. The cell of analysis (made of platinum) was filled (600 μL). A pressure of 3 atm was used, with a heating rate of 1 °C.min^−1^ in an analysis range of 0 °C to 100 °C. All samples were degassed immediately before use.

### 2.9. Mucoadhesion Characterization Tests

#### 2.9.1. In Vitro Mucoadhesion Test

This method was adapted from [23,24]. Lyophilized nanoparticles (20 mg) were added to 10 mL of mucin aqueous solutions at different concentrations (50, 100, 150, and 200 g/mL), vortexed, and incubated in the thermostatic bath at 37 °C for 1 h. Then, they were centrifuged at 8000 rpm (5 min), and the supernatant was used for the quantification of free mucin (749 nm), using a Lowry protein assay modified by Peterson [24,25,26]. Free mucin determination from the supernatants was quantified using the Total Protein Kit, Micro Lowry, and Peterson’s Modification (Sigma-Aldrich^®^, St Louis, MO, USA) were used. Data from mucin adsorption were fitted using Freundlich (Equation (2)) and Langmuir (Equation (3)) models that describe the adsorption isotherms [25].

(2) $$Q_{e}=KC_{e}C_{e}^{1/n}$$

(3) $$\;\;\;\;Q_{e}=\frac{aC_{e}}{b+C_{e}}$$

*Q_e_* is the amount of mucin adsorbed on nanoparticles (mg.g^−1^) per unit mass, and *C_e_* is the concentration of free mucin (mg.L^−1^), at equilibrium. For the Langmuir equation, 1/*Q_e_* was plotted against 1/*C_e_* to obtain the constants b (dissociation coefficient) and a (binding capacity). Log *C_e_* was plotted against *C_e_* to obtain the constants 1/*n* (adsorption capacity) [23,24,25].

#### 2.9.2. Ex Vivo Mucoadhesion Test

The ex vivo mucoadhesion test was performed according to the procedure proposed by [26], with some modifications [25]. Fresh porcine large intestine tissue was washed with saline solution (0.9%) and opened longitudinally. Pieces (5 cm × 3 cm) were attached to the inclined plastic support (90°) of the device. Nanoparticle suspensions (40 µL) were placed gently on the surface of te tissue, and a 30 s contact time with the mucous layer was allowed. After this, the tissue was rinsed with simulated mucous at a rate of 1 mL/min for 5 min using a peristaltic pump, and the fluid was collected. The MTX was quantified in the collected fluid, so the non-adsorbed fraction of the drug on the mucosal surface could be determined. The fluid was alkalinized with NaOH 0.1 M, pH = 10, for nanoparticle destruction and drug release, centrifuged at 14,000 rpm for 30 min, filtered through a 45 µm membrane, and injected into HPLC. The test was performed in triplicate for the NPPM29 and MTX solution. Simulated mucus was composed of 2% (*w*/*v*) of mucin, 0.745% (*w*/*v*) of NaCl, 0.129% (*w*/*v*) of KCl, and 0.032% (*w*/*v*) of CaCl_2.2_H_2_O. The mixture was stirred under magnetic stirring until complete dispersion, and the pH was adjusted to 5.7 with NaOH 0.1 M [27].

### 2.10. In Vitro Drug Release Test

Franz diffusion cells were used (Hanson Research^®^, Cotia, Brazil), filled with 7.0 mL of receptor fluid. A synthetic cellulose acetate membrane with a molar mass cut-off of 12–14 kDa (Spectrum) was previously treated with Milli-Q water at 100 °C for 15 min and placed on a 1.77 cm^2^ area. The experiment was carried out for an acidic dissolution medium composed of chloridric acid buffer 0.1 M, pH = 1.2, during 1 h, as well as for neutral pH composed of NaOH solution 0.1 N, buffered with 0.9% sodium chloride, pH = 7.5, during 6 h. All experiments were performed at 37 °C at 300 rpm, and sink conditions were maintained. Samples of 1 mL were withdrawn from the receptor fluid, and the volume withdrawn was replaced with fresh receptor fluid. Each formulation was analyzed in triplicate, containing MTX at 0.120 mg.mL^−1^. MTX was quantitatively determined by HPLC at 306 nm using a UV–Vis detector.

The drug release kinetics were evaluated by different mathematical models, such as zero order, first order, Weibull, Korsmeyer–Peppas, and Higuchi [28]. To select the best model that fits the release profile obtained for the samples, the correlation coefficient (R) was used. The kinetic drug release models can be plotted using Sigma Plot software, which presents a library of most employed mathematical models used for controlled drug release systems.

### 2.11. In Vitro Assays

#### 2.11.1. Cell Lines and Culture Conditions

This study utilized the U251MG (glioblastoma) and CCD 1059Sk (primary skin fibroblasts) cell lines, obtained from the Rio de Janeiro Cell Bank. The cells were cultured in DMEM (Dulbecco’s Modified Eagle Medium, Sigma, Saint Louis, MO, USA) supplemented with 10% fetal bovine serum (FBS, Cultilab, Campinas, SP, Brazil) and maintained in an incubator at 37 °C with 5% CO_2_. Subculturing was performed every two days, and cell stocks were stored in liquid nitrogen.

#### 2.11.2. Cell Viability Study

Cell viability was assessed using the MTS assay (3-(4,5-dimethylthiazol-2-yl)-5-(3-carboxymethoxyphenyl)-2-(4-sulfophenyl)-2H-tetrazolium) (Promega Corporation, Madison, WI, USA). The experiments were conducted in duplicates, and the results obtained from the experimental series were confirmed in a subsequent experiment. The *IC*_50_ value (the concentration capable of inhibiting 50% of cell growth) was calculated using GraphPad Prism 8^®^ (GraphPad Software, Inc., San Diego, CA, USA).

#### 2.11.3. Evaluation of Cellular Internalization by Fluorescence Microscopy and Laser Scanning Confocal Microscopy Analysis (LSCM)

U251MG cells were seeded on 35 mm diameter plates with coverslips at a density of 2 × 10^5^ cells per plate. After adherence, the cells were exposed to the compounds of interest for varying periods, depending on the experimental approach. At the end of the incubation period, the cells were fixed with 3.7% formaldehyde for 30 min, and the nuclei were counterstained with DAPI (4′,6-Diamidino-2′-phenylindole dihydrochloride, Molecular Probes, Life Technologies, Carlsbad, CA, USA). After washing, the slides were mounted with VectaShield^®^ (Vector, Newark, CA, USA) and analyzed using a fluorescence microscope (Eclipse 80i, Nikon, Melville, NY, USA) or a laser scanning confocal microscope (C2 Plus, Nikon).

#### 2.11.4. Flow Cytometry Analysis

U251MG cells were seeded on 35 mm diameter plates at a density of 2 × 10^5^ cells per plate. After adherence, the cells were exposed to the compounds of interest for different periods, depending on the experimental approach. At the end of the incubation period, the cells were harvested by enzymatic digestion (trypsin/EDTA, 1:250, Sigma). The cell suspension was centrifuged (5 min at 1000 rpm), and the cell pellet was resuspended in a culture medium (200 µL). The analysis was immediately performed using a flow cytometer (Guava easyCyte 8HT, Merck/Millipore, Saint Louis, MO, USA) with InCyte software (Merck/Millipore, Saint Louis, MO, USA). The samples were analyzed by flow cytometry at 488/520 nm excitation/emission wavelengths.

### 2.12. Preparation of QS Solution Conjugated with FITC (Fluorescein 5(6)-Isothiocyanate)

To prepare a QS solution conjugated with FITC, 5 µL of FITC solution in methanol (0.01 g/mL) was added to 1 mL of QS solution (1.82 mg/mL) in 0.1 N acetic acid. The mixture was vortexed and stored in the dark for 8 h. The pH of the solution was adjusted to 10 using 0.5 M NaOH; then, it was centrifuged for 30 min at 14,000 rpm. The supernatant containing excess FITC was discarded. The unreacted FITC was washed by resuspending the QS in water and centrifuging (10 min at 14,000 rpm); this process was repeated two times. After discarding the aqueous supernatant, the precipitated QS was resuspended in 1 mL of 0.1 N acetic acid and vortexed.

The resulting solution was placed in a dialysis membrane and immersed in water at room temperature, with agitation at 200 rpm for 15 min to continue removing unreacted FITC. The water in the beaker was changed four times, and the absence of fluorescence in the wash water was verified using a fluorometer. If necessary, the volume of the QS-FITC solution was adjusted to 1 mL, the pH was adjusted to 5.5, and NPP29 (empty) was prepared (adapted from [29]). The internalization profile in NPP29 was analyzed by flow cytometry and fluorescence microscopy. Cultures were exposed to NPP29 for 24 h (25 µL of stock solution in 1 mL of culture medium), and the samples were subsequently analyzed.

### 2.13. Comparative Analysis of MTX Conjugated with FITC Internalization Profile

The comparative analysis of MTX/FITC and NPPM29 (MTX/FITC) internalization was performed in U251MG cultures exposed for 2 and 6 h. A subsequent test was conducted to evaluate the retention potential of MTX/FITC in the presence of polymers (QS and HPMCP) at 12 and 24 h. A new experimental group was tested, including verapamil (10 µM) as a positive control. For this, verapamil was used in a 24 h pretreatment [30], and the cells were exposed to the compounds of interest for 2 h. The analysis was performed by flow cytometry.

### 2.14. Statistical Analysis

The results were presented as the average value of three replications with the respective standard deviations. One-way analysis of variance (ANOVA) followed by the Dunnet test was used to evaluate significant differences. A significance level of 5% was adopted.

## 3. Results and Discussion

The mean diameter results of nanoparticles are in Table 2. The smallest particle was produced with TPP (NPTM33); however, its zeta potential presented the lowest value, which resulted in less stability of the sample. High values of zeta potential result in more stable suspensions because the repulsion between the particles avoids aggregation [30]. Although TPP nanoparticles showed a considerably smaller size (145.40 nm), they presented greater turbidity when compared with nanoparticles with HPMCP. The size increment of NPP-type nanoparticles can be due to the high volume of the polymeric chain of HPMCP. Statistical analysis comparing the NPP-type group of nanoparticles indicated NPPM26 and NPPM29 as the smallest ones, giving average values of 456.00 nm and 452.60 nm, respectively (*p* > 0.05). Both have in common the lowest proportion difference between chitosan and HPMCP (3:1). The difference between them is the increase of 10× of the loaded MTX in NPPM29 compared to NPPM29. This result suggests the optimum polymer proportion is 3:1 (QS: HPMCP), and the increase in the drug loading did not interfere with the nanoparticle’s diameter.

Polydispersity index (PDI) results are disclosed in Table 2. The values were similar for all nanoparticles (*p* < 0.05) and ranged from 0.22 to 0.34. PDI values smaller than 0.5 indicate size distribution homogeneity [31].

Zeta potential results are in Table 2, and the obtained values were high and positive, ranging from 15.90 to 25.83 mV. NPTM33 presented the lowest value, which can result in low stability. High values of zeta potential result in more stable suspensions because the repulsion between the particles avoids aggregation [31]. This result corroborates the visual observation of a higher turbidity for TPP nanoparticles than for HPMCP nanoparticles.

The loading efficiency (*LE*%) can be seen in Table 2. Nanoparticles containing 0.05% MTX (NPPM 23 to 25) and 0.5% MTX (NPPM 26 to 28) resulted in very low *LE*%. Samples NPPM29 and 30 containing 5% MTX showed the highest values of *LE*%, incorporating almost the most amount of drug added during nanoparticles’ production. Further addition of MTX, such as 10% for NPPM34 and 20% for NPPM35, resulted in precipitation of the dispersion. Despite NPPM30 having the highest LE% (92.71%), there was no significant difference between NPPM29 (91.72%). Since the latter resulted in a lower size, it was selected for further studies.

**Figure 1:**
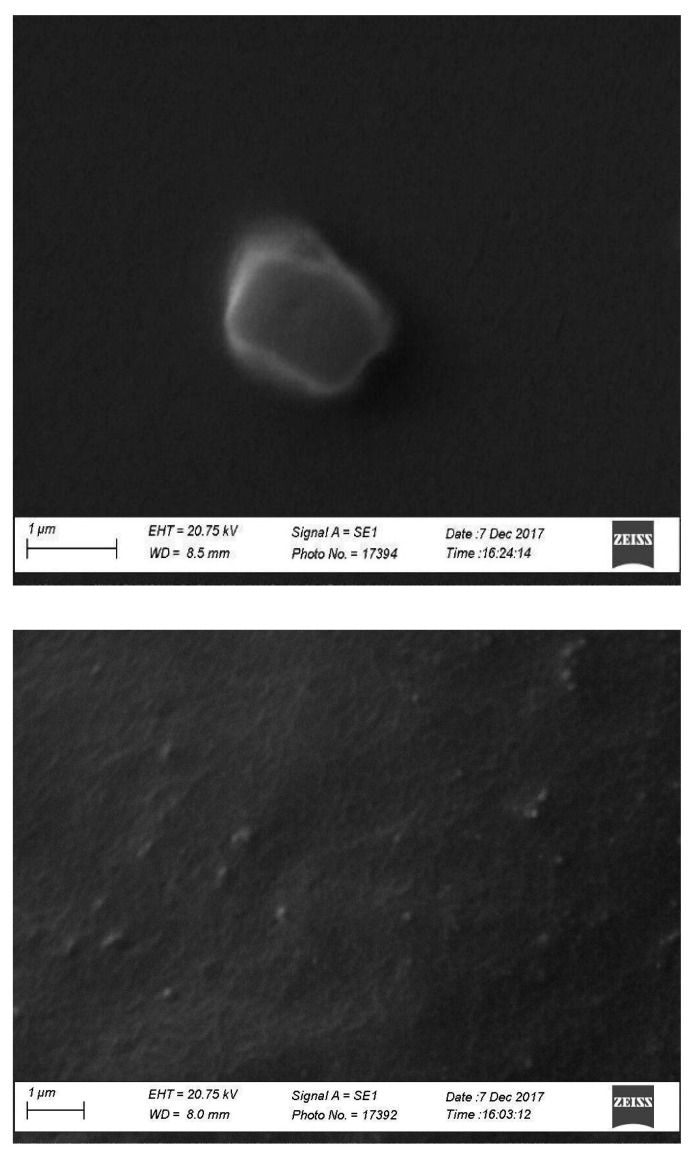
SEM microphotograph of NPPM29 at 32,830× (**top**) and 20,000× (**bottom**).

The morphology of the NPPM29 is shown in Figure 1. The MTX-loaded nanoparticles were nearly geometrical in shape, due to the presence of MTX presenting a needle-like structure, which changed the typical spherical shape of chitosan nanoparticles. The morphology result corroborates with the X-ray diffraction spectrum of NPPM29, showing the maintenance of MTX crystallinity in the nanoparticles.

### 3.1. FTIR Spectra

The FTIR spectra of all samples are disclosed in the Supplementary Materials. The chitosan spectra exhibited bands between 3460 and 3295 cm^−1^ assigned to the stretching vibration of –OH overlapping -NH, bands at 1647, 1582, and 1377 cm^−1^ related to -(CO)-NH_2_, NH_2,_ and C-N [32]. TPP presented characteristic bands of phosphate groups at 1122 cm^−1^ and P-O-P groups at 885 cm^−1^ [33]. HPMCP presented the bands of stretching vibration of –OH between 3650 and 3400 cm^−1^, C-H bond (sp3) at 2923 cm^−1^, C=O group at 1735 cm^−1^, C-O ether bond at 1217 cm^−1^, C-O ester bond at 1058 cm^−1^, and monosubstituted aromatic ring at 742 cm^−1^ [23,34,35]. It can be seen in the MTX FTIR spectra that the primary aliphatic amines band is at 3329 cm^−1^, the carboxylic acid (O-H) bands are between 3170 and 2780 cm^−1^, and the C=O band is at 1670 cm^−1^. The stretching of the amide group (CONH) occurred at 1641 cm^−1^, bands corresponding to the aromatic ring (C=C) can be seen at 1535, 1490, and 1413 cm^−1^, and the secondary amine C-N band is at 1402 cm^−1^ [36].

For nanoparticles, it was observed that the characteristic bands of chitosan at 3460–3295 cm^−1^ were displaced and increased, indicating a complexation of chitosan by hydrogen bonds [37]. The band changes can be correlated with the chemical interactions of QS and MTX of the NPTM33 and NPPM29. The interaction of drugs and polymeric matrices is another important interaction. The amide formation can be confirmed with the band at 1641 cm^−1^ (Supplementary Materials).

The FTIR spectra of NPTM33 (Supplementary Materials) show a broadening of the band between 1647 and 1377 cm^−1^, corresponding to the C=O and C-N of chitosan [38], in addition to the displacement of the 1122 cm^−1^ band related to (P-O) group of TPP. These modifications can be attributed to the interaction between chitosan and TPP.

For the FTIR spectra of NPPM29 (Supplementary Materials), the C=O peak of HPMCP at 1735 cm^−1^ is absent, and there is a displacement of bands at 1647 and 1582 cm^−1^ attributed to (-CO)NH_2_ and NH_2_ groups of chitosan, which can be due to interaction with the carbonyls of HPMCP. In addition to that, a new band was detected at 1548 cm^−1^, which suggests a polyelectrolyte interaction [23,32].

Comparing the NPTM33 and NPPM29, changes in the characteristics of the bands 3460–3295 cm^−1^ related to the overlapping OH stretching of NH, can be attributed to an interaction between chitosan and MTX that is less evident in NPPM29 suggesting a higher interaction of these groups.

### 3.2. PXRD Analysis

**Figure 2:**
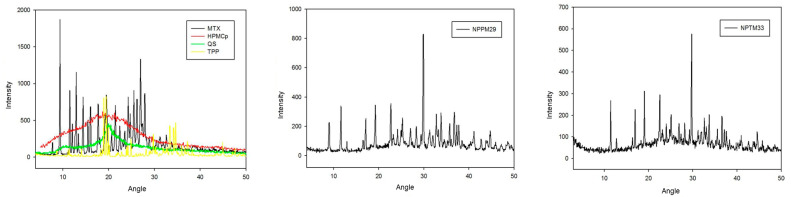
X-ray diffraction spectrum of the drug and polymers and NPPM29 and NPTM33.

The X-ray diffraction spectrum MTX peaks of crystallinity are in Figure 2. Up to about 40° (2θ), mainly in the regions between 8° and 30° (2θ), being the one with the highest intensity and characteristic at 9.3° (2θ) [39].

HPMCP X-ray diffraction spectrum (Figure 2) showed no crystallinity peaks, characteristic of amorphous polymers [35]. For chitosan, it was observed that two peaks around 10° and 20° (2θ) can be correlated to the anhydrous crystals [40]. For TPP (Figure 2), peaks of crystallinity between 10° and 40° (2θ) [34].

The X-ray diffraction spectra of NPPM29 and NPTM33 are shown in Figure 2. The presence of two new peaks of crystallinity at 2θ can be observed, which indicates that the complexation between the polymers could cause a structural rearrangement leading to the formation of new structures with crystalline characteristics [23,32].

Diffraction peaks of the components are detectable on the PXRD patterns of the nanoparticles. Several distinctive sharp peaks in the diffractogram of MTX-loaded nanoparticles indicate the high degree of drug crystallinity in the NPPM29 and NPTM33 systems physical mixture.

### 3.3. Thermal Analysis Results

**Figure 3:**
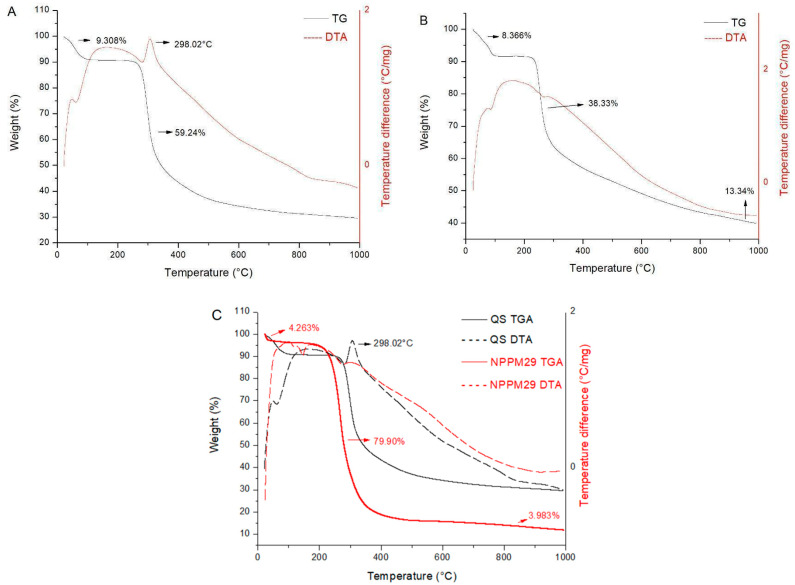
TGA and DTA curves of QS (**A**), MTX (**B**), and QS compared to NPPM29 (**C**) obtained in the 25 °C range at 1100 °C, under a nitrogen atmosphere and heating rates of 20 °C.min^−1^.

The TGA curves are in Figure 3. For chitosan, there is a first event at 56.80 °C characteristic of dehydration with 9.308% of weight loss between 40 and 120 °C; then, the second event at 250 °C characterizes the polymeric degradation.

DTA curves (Figure 3) show a valley at 56.80 °C and 298.02 °C, initiated by an endothermic process followed by an exothermic event, indicating the thermal degradation of chitosan. MTX and nanoparticles presented weight loss due to dehydration followed by decomposition. Particularly in the case of NPPM29, the peak corresponding to dehydration was displaced to 140 °C in the DTA curve, and the peak at 298.02 °C of chitosan degradation is absent, indicating a possible increase in the thermal stability of the nanoparticles.

**Figure 4:**
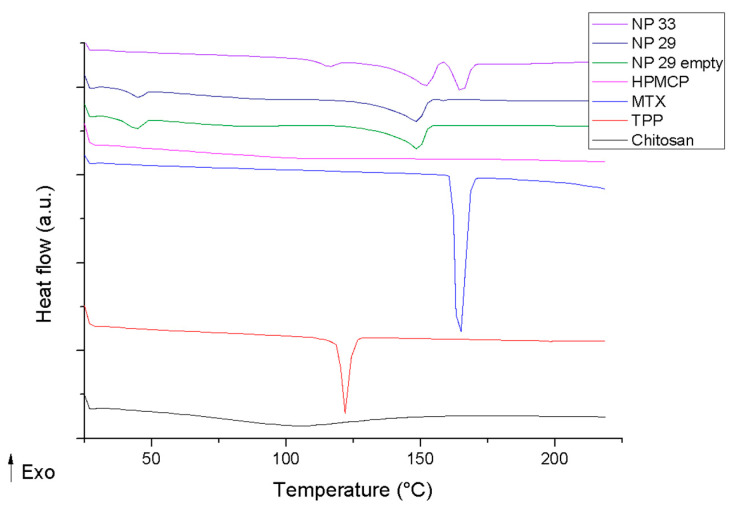
DSC curves of methotrexate (MTX), nanoparticles (NPP29, NPPM29, NPT33, NPTM33), and polymers (chitosan, HPMCP, and TPP).

Figure 4 demonstrates that NPPM29 with and without the drug shows the same exothermic events. A drug protection effect can be suggested since there is no peak of MTX melting. For NPTM33, it was possible to observe the drug melt event at 170 °C, demonstrating that a fraction of free drug can be present; the addition of TPP prejudices the interaction of the drug with the system. It is suggested that HPMCP, due to its smaller size and more electronegative than MTX, interacts more strongly with chitosan, displacing MTX bonds from chitosan.

NPs have emergent events that were not seen in the isolated materials. This reinforces the presence of an interaction between chitosan and HPMCP, forming a new supramolecular structure.

The heat capacity (Cp) acts as the identity of the material and is a precise value. Any change in this value indicates changes in the material structure. Systems where supramolecular bonds are strong and oriented demonstrate high Cp.

The NPs have a distinct Cp value from the isolated components, reinforcing the interaction and structuring of the drug delivery system. NPPM29 Cp (7.43 J/g K) has close values to unloaded NPP29 (7.77 J/g K). This is corroborated by the DSC data, showing the drug is encapsulated in the drug delivery system. NPTM33 has lower Cp (5.56 J/g K) than NPPM29, suggesting that TPP disorganizes the supramolecular bonds, and disassembles the system.

**Figure 5:**
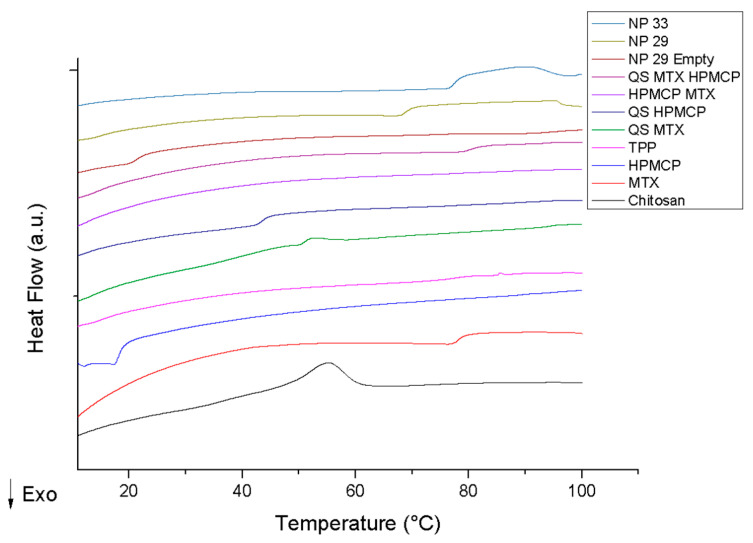
NanoDSC curves of methotrexate (MTX), nanoparticles (NPP29, NPPM29, NPT33, NPTM33), and polymers (chitosan, HPMCP, and TPP).

The results of nanoDSC, unlike the DSC, enable us to demonstrate how water influences molecular interactions. As shown in Figure 5, the TPP has not had any events; the curve of HPMCP and MTX shows a baseline shift. The chitosan event can be attributed to a breakdown of hydrogen bonds of the polymer dispersed in water.

The mixture between chitosan and HPMCP proves the effective interaction between the polymers, shown by the absence of the chitosan event and the HPMCP event being moved to a higher temperature. The physical mixing of chitosan with MTX demonstrates that MTX attenuated the chitosan event. The carboxyl groups of MTX can interact by electrostatic bonds with chitosan. As an extensive polymer, the number of MTX molecules may not be enough to fully interact with chitosan, which results in an attenuated event. The mixture between HPMCP and MTX did not present any events; both are anionic agents, and an electrostatic repulsion between the excipients is expected.

NPTM33 exhibits a unique endothermic event; the nanoparticle undergoes a rupture of the supramolecular bonds, leading to the separation of the polymer chains.

The NPP29 (empty) event suggests a free HPMCP fraction in the solution. NPPM29 loaded with the drug this event is not visualized; the drug can promote a greater interaction between these free portions. Also, the loaded NPPM29 exhibits an event at a higher temperature, suggesting that the drug is encapsulated and corroborates the physicochemical stability of the drug delivery system.

Therefore, thermal analysis was important to indicate a stronger interaction of chitosan with HPMCP than TPP and the higher protection and incorporation of MTX in the nanostructure.

### 3.4. Mucoadhesion Characterization Results

**Figure 6:**
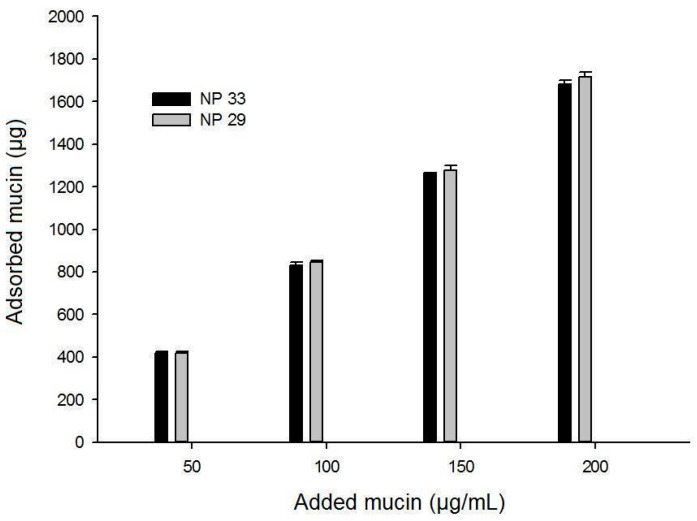
Adsorbed mucin on nanoparticles related to the amount of added mucin.

The relation between the amount of adsorbed mucin and added mucin is demonstrated in Figure 6. For nanoparticles NPTM33 and NPPM29, the quantity of adsorbed mucin was proportional to the added mucin, without a significant difference (*p* > 0.05). Table 3 shows the values of r^2^ that also presented similar values among Langmuir and Freundlich models. The b-values were analogous for both nanoparticles, but NPPM29 presented a higher value of a (binding capacity) than NPTM33, which can be due to its greater zeta potential, resulting in better affinity to the mucin. When the variable 1/n assumes values smaller than 1, it is indicative that the electronic interaction is stronger than hydrogen or Van der Waals bonds [23]. Therefore, these models indicate the mucoadhesion mechanism that prevails for nanoparticles and mucin is electrostatic interactions, and HPMCP contributed to an increase in the adsorption compared to TPP.

Using a falling liquid apparatus, it was found that 84% *w*/*v* of MTX-loaded NPPM29 stayed on the mucosa, while 10.5% of MTX solution remained on the mucosa. This result, combined with the previous in vitro mucoadhesion study, shows how chitosan/HPMCP nanoparticles potentially avoid the rapid elimination caused by mucociliary clearance, in addition to allowing longer contact with the target site.

### 3.5. In Vitro Drug Release Results

**Figure 7:**
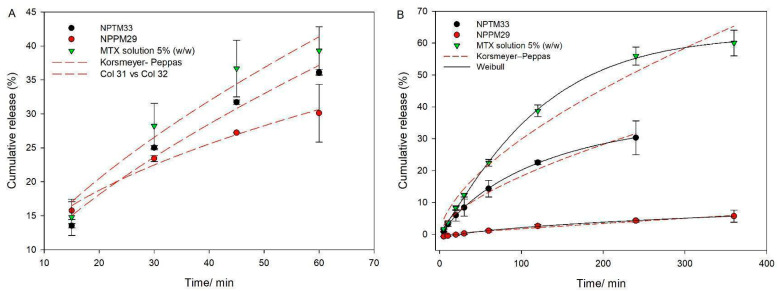
Drug release profile of NPTM33, NPPM29, and MTX solution in acidic (**A**) and neutral environments (**B**). The lines indicate the fit of the Korsmeyer–Peppas and Weibull kinetic models.

The percentage of MTX release against time is represented in Figure 7. The drug release profiles were fit by mathematical models designed for drug release mechanism identification. Several models are described in the literature, and the criterion used to choose between these models is the coefficient of correlation (R). Korsmeyer–Peppas and Weibull models were better adjusted since R-values were greater than 0.97.

It is possible to obtain the release exponent n from the Korsmeyer–Peppas model that characterizes the drug release mechanism. For n ≥ 0.89, the drug release follows Fickian diffusion); n ≤ 0.45 indicates a case II transport, controlled by relaxing or swelling of polymeric chains, and 0.45 < n < 0.89 means a combination of the mechanism of diffusion and case II [28,35]. Results indicate the drug release in an acidic environment is a case II transport for NPPM29, since n = 0.45. The n exponent was 0.8 for a neutral environment, so an association of mechanisms was found for NPPM29. Chitosan precipitates under neutral pH, while HPMCP ionizes and swells. So, MTX may diffuse through the gel formed by HPMCP, and the erosion of chitosan modifies the drug release. Nanoparticles composed of TPP (NPTM33) followed the case II mechanism, independent of the pH of the release medium (n = 0.65 for pH 1.2 and n = 0.63 for pH 7.4). Since TPP is an ion, the pH influences the release profile. The chitosan swelling in acidic pH did not control MTX release, while in neutral pH its precipitation prolonged the drug release by erosion.

From the Weibull model, it is possible to obtain the curve shape parameter that characterizes the curve as it is used to predict the drug release model. Values of b < 0.75 indicate Fick diffusion; values between 1 > b > 0.75 are associated with Fickian diffusion and case II (swelling), and the b > 1 result represents that a complex mechanism governs the release process [41]. The power of the test for fitting the drug release profile in an acidic environment was not satisfactory, so only the neutral release medium was evaluated. The release profile of NPTM33 followed diffusion and case II mechanisms (n = 0.63), while for the drug release of NPPM29, n = 1.2 suggests more complex mechanisms are involved. These results confirmed the suggestion of polymer performance to control the MTX release. The modification of drug release of NPTM33 is due to erosion of the chitosan matrix precipitated in neutral pH, while for NPPM29, two polymers are performing at the same time, and the MTX release is dependent on more mechanisms: erosion of chitosan and swelling of HPMCP, resulting in a considerably prolonged MTX release in neutral pH [38]. These results correlate with FITR analysis since HPMCP is a longer-chain polymer than TPP; there are more sites for electrostatic interaction with chitosan. This interaction may also contribute to more prolonged retention of the drug during drug release [42].

Considering the biological environments for drug release, in the case of the oral route, the particles did not promote gastro resistance in acidic pH, so other strategies may be explored for controlling drug release, such as a gastro-resistant coating. In the case of other mucosal routes, where the pH is close to neutrality, such as nasal, ocular, vaginal, and rectal, a prolonged drug release may take place, and nanoparticles composed of HPMCP showed great improvement when compared to conventional chitosan nanoparticles composed of TPP.

### 3.6. Cell Viability Results

The relative cell viability was obtained with the MTS assay using U251MG glioblastoma cell line and CCD 1059Sk fibroblasts treated for 48 h with NPs at different concentrations.

**Figure 8:**
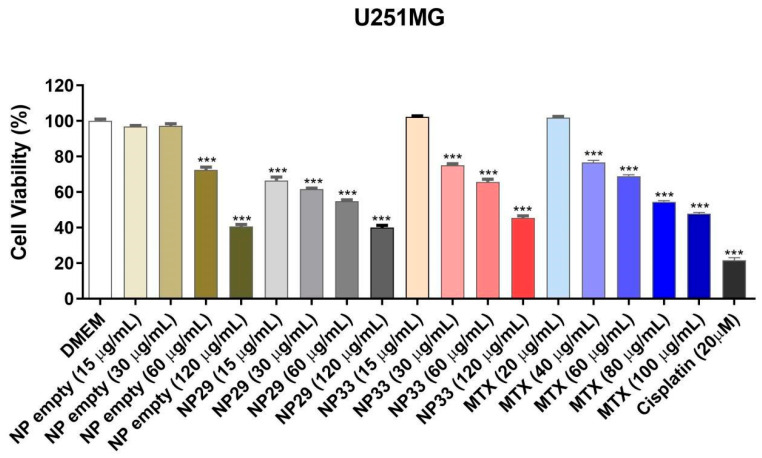
Relative cell viability of U251MG glioblastoma cells determined by MTS assay. (NPP29) CS/HPMCP nanoparticles, (NPPM29) CS/HPMCP/MTX nanoparticles, (NPTM33) CS/TPP/MTX nanoparticles, and MTX (Methotrexate). (***) *p* value ˂ 0.001 according to ANOVA followed by Dunnet’s post-test.

A reduction in cell viability for the unloaded particle (NPP29) and MTX was observed at 60 µg/mL and 40 µg/mL, respectively. However, the drug-loaded NPs (NPPM29 and NPTM33) showed a reduction in viability at the lowest concentrations used, 15 µg/mL (Figure 8).

Based on these data, the *IC*_50_ was calculated (Table 4), showing that NPP29 had low cytotoxicity in the glioblastoma cell line, requiring 94.81 µg/mL to inhibit 50% of cell growth. For the drug-loaded nanoparticles (NPPM29 and NPTM33), *IC*_50_ values of 68.79 and 74.55 µg/mL were obtained (Table 4), indicating higher toxicity to glioblastoma cells than the free drug. The NPPM29 sample exhibited the highest cytotoxicity, requiring a lower concentration to reduce cell viability by 50%. The values found for the NPs are close to those found by Mangaiyarkarasi (2015), of 71.2 µg/mL, in a study with CS-conjugated LaF3:Tb3+ NPs containing MTX in tumor cells.

These findings suggest a higher specificity of HPMCP nanoparticles to U251MG glioblastoma cells than the free-MTX or TPP nanoparticle.

**Figure 9:**
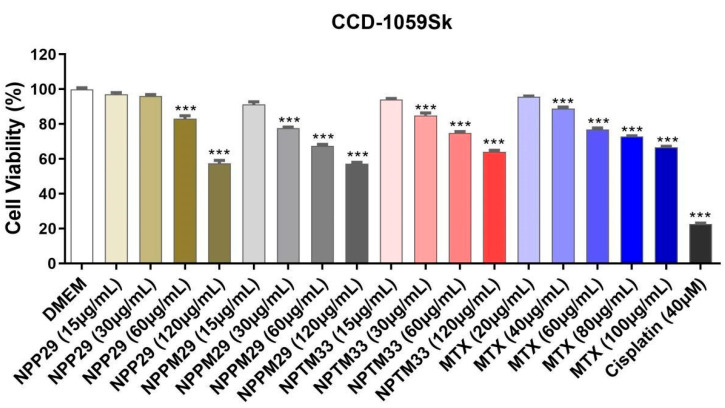
Relative cell viability of primary skin fibroblasts (CCD-1059Sk) determined by MTS assay. (NPP29) QS/HPMCP NPs, (NPPM29) QS/HPMCP/MTX NPs, (NPTM33) QS/TPP/MTX NPs, and MTX (Methotrexate). (***) *p* value ≤ 0.001 according to ANOVA followed by Dunnet’s post-test.

In tests conducted with fibroblasts (Figure 9), it was observed that the highest concentration used did not render 50% of the fibroblast cells non-viable, making the *IC*_50_ calculation impossible. Therefore, no significant reduction in cell viability was noted, indicating that the tested NPs exhibited low toxicity toward fibroblast cells.

### 3.7. Flow Cytometry, Fluorescence Microscopy, and Laser Scanning Confocal Microscopy (LSCM)

The internalization profile of MTX and NPPM29 was analyzed using flow cytometry and confocal microscopy. Flow cytometry allows multiparametric analysis of individual cells (in suspension) concerning their physical or chemical characteristics. The equipment is equipped with various lasers that serve as illumination sources, enabling the quantitative determination of cells that have been previously labeled with fluorophores [43]. Flow cytometry has been widely used in experimental approaches to evaluate the internalization capacity of drugs and the drug’s ability to inhibit the efflux pump mediated by P-glycoprotein (P-gp) [44,45].

P-gp is a glycoprotein that belongs to the structure of the cell’s membrane that actively pumps out its substrate after it has been internalized by the cell, a phenomenon closely related to drug resistance mechanisms. If P-gp substrates are labeled with fluorophores, an increase in the frequency of fluorescent cells can be detected by flow cytometry in response to agents capable of inhibiting P-gp. Thus, a quantitative analysis based on the fluorescence intensity detected by the equipment is possible [45].

This study evaluated the anti-efflux activity through the cellular internalization of the formulation and its association with MTX of the polymers involved in its preparation. These compounds were compared to the free drug (MTX/FITC). Verapamil, a known substrate of the P-gp efflux enzyme, was used as a positive control [28,45].

**Figure 10:**
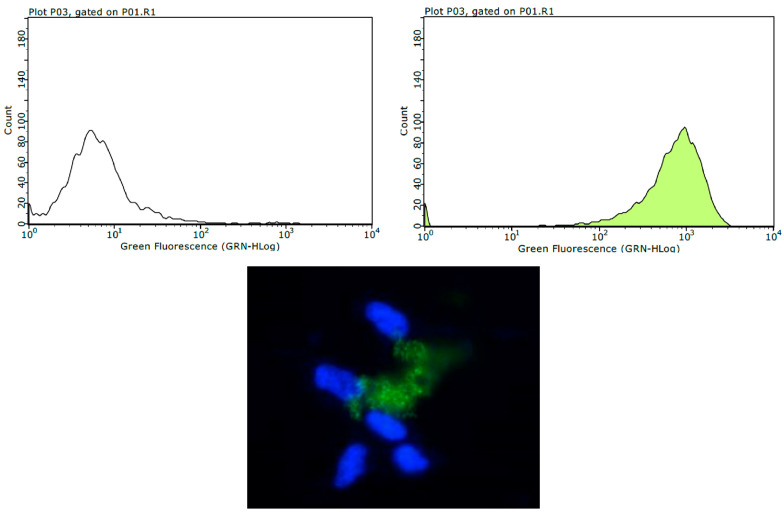
Incorporation profile of NPP29 (QS-FITC) by U251MG cells, derived from glioblastoma, by flow cytometry and fluorescence microscopy. In (**top**, **left**) flow cytometry analysis of control cells (not exposed to NPP29); (**top**, **right**) flow cytometry analysis of cells exposed to NPP29 for 24 h; (**bottom**) illustrative image obtained by fluorescence microscopy (400×) evidencing positive cells, which internalized the NPP29 (QS-FITC, green). The cell nuclei were marked with DAPI and are evidenced in blue.

The empty NPs were evaluated to observe the effect of the developed particulate system without MTX (NPP29) on U251MG cells. QS was labeled with a fluorescent agent (FITC) to allow the evaluation of the incorporation profile of this polymer without the drug. The results showed an increase in the frequency of FITC-positive cells after 24 h of exposure, as evidenced by the rightward shift in the peak on the fluorescence intensity axis (Figure 10). Observations made using fluorescence microscopy also revealed the presence of positive cells, indicating efficient incorporation of NPP29 by the cells (Figure 10).

**Figure 11:**
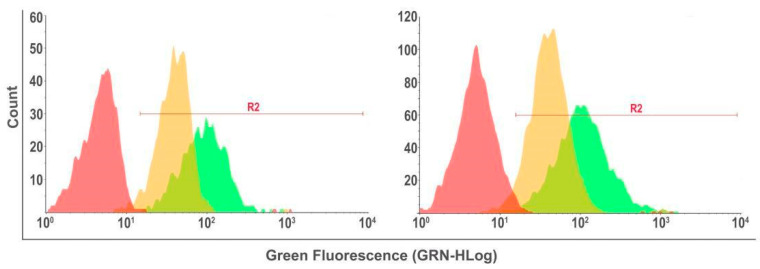
Flow cytometry results of glioblastoma cells incubated with MTX/FITC and NPPM29 for 2 h (**left**) and 6 h (**right**) exposure. Fluorescence intensity profiles: red population: exposed to culture medium only (negative control), yellow population: exposed to MTX-FITC, green population: exposed to NP-FITC.

Loaded nanoparticles were evaluated using 25 µL of MTX/FITC, and NPPM29 samples were used in 1000 µL of culture medium, with cultures exposed for 2 and 6 h. The 2 h time was considered more promising and was therefore used in subsequent experiments investigating the effect of MTX/FITC, NPPM29, and verapamil on the efflux pump. From the results shown in Figure 11, the cell percentage that incorporated the NPs after 2 h of exposure was 96.9%. This percentage was higher than that found for the cells treated with free MTX/FITC, which was 91.7%. Thus, it was observed that the NP internalization profile was efficient in U251MG cells.

**Figure 12:**
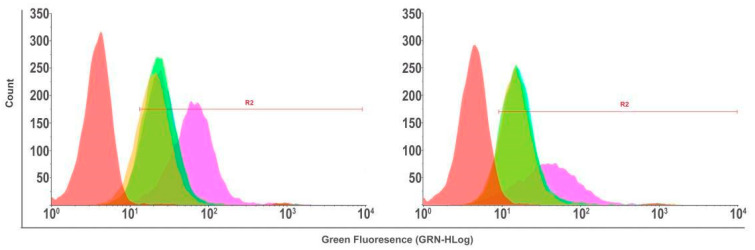
Flow cytometry analysis on U251MG cells, derived from glioblastoma, exposed to MTX/FITC (orange), MTX/FITC-QS (green), MTX/FITC-HPMCP (light blue), and NPPM29 (pink) for 12 h (**left**) and 24 h (**right**). Fluorescence intensity profiles: The red range represents the population exposed only to the culture medium, thus without fluorescence (negative control).

The effect of the isolated polymers can be seen in Figure 12, which compares the different polymers with MTX/FITC and NPPM29. After 12 h of treatment, the associations of both QS and HPMCP with MTX/FITC promoted approximately 8% more internalization of the drug than the free form, indicating their activity on the P-gp efflux pump. NPPM29 exhibited 97% fluorescence, while MTX showed 81.21% after 12 h. After 24 h, fluorescence decreased by 13.8% for the NPs and 30.33% for MTX, indicating that a larger quantity of NPs was internalized and retained longer within the cells.

**Figure 13:**
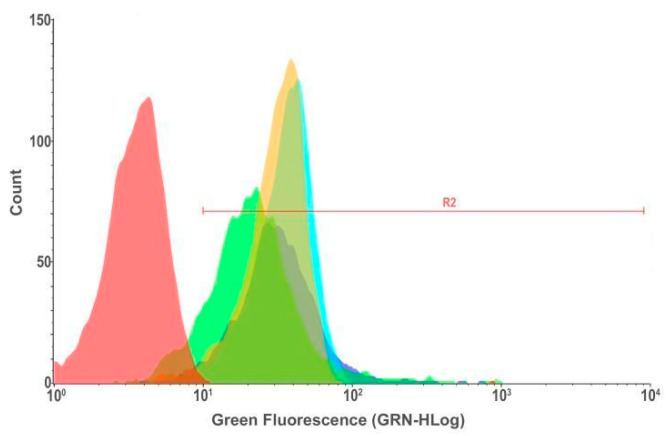
Flow cytometry analysis of U251MG glioblastoma cells exposed to MTX/FITC (green), NPPM29 (orange), MTX/FITC-verapamil (10 µM) (dark blue), and NPPM29-verapamil (10 µM) (light blue) for 24 h. The red interval represents the population exposed only to the culture medium, without fluorescence (negative control).

The pretreatment with verapamil (10 µM) was conducted for 24 h, followed by a 2 h treatment of the cultures with the different compounds [28]. As shown in Figure 13, the anti-efflux activity of NPPM29 was confirmed, as the frequency of positive cells was higher in cultures treated with the drug associated with verapamil than the drug alone.

**Figure 14:**
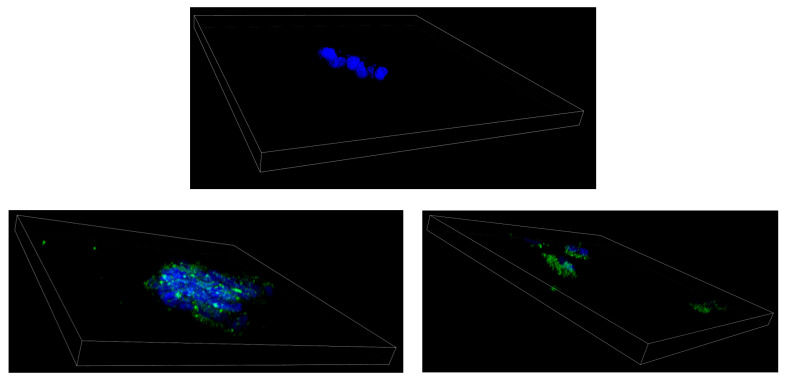
Three-dimensional Images Generated by Confocal Microscopy of NPPM29 Inside Tumor Cells After 12 h (**bottom-left**) and 24 h (**bottom-right**) of Treatment. Cells without treatment as control (**top**). The green fluorescence corresponds to the FITC in the NPs.

The cellular internalization of NPPM29 was confirmed by confocal microscopy, where intense green fluorescence from FITC in the NP was observed in the cytoplasm around the nucleus (blue) (Figure 14). This observation confirms the specific and efficient action of the NP as a drug delivery system, particularly after 12 h of treatment.

Flow cytometry associated with the confocal microscopy experiments exhibited significantly superior cellular internalization, as the NPs, regardless of the exposure time, not only showed higher selectivity for cancer cells but also exhibited enhanced drug delivery performance, maintaining a greater amount of MTX within the cells in a sustained manner.

## 4. Discussion

MTX is widely explored in the treatment and control of chronic severe and high-incidence diseases such as cancer, psoriasis, and rheumatoid arthritis; however, this drug is still a challenge to pharmaceutical sciences due to its toxicity, resistance, and low bioavailability limitations.

This study focused on investigating a potential tumor-specific and P-gp efflux inhibition activity of chitosan- and HPMCP-based nanoparticles to target drug delivery for cancer cells as an alternative to reduce the dose and side effects.

Nanoparticles were successfully developed using the polyelectrolyte complexation method, a simple and cost-effective technique suitable for bench-scale laboratory for in vitro proof of concept study applications. The polyelectrolyte interaction between chitosan and HPMCP could be evidenced by the FTIR spectra, DSC, and nanoDSC curves, confirming the supramolecular interaction due to the polyelectrolyte crosslink process. Nanoparticles were geometrically in morphology, containing the MTX in a crystalline form, confirmed by the PXRD analysis. Chitosan/HPMCP nanoparticles showed superiority in the in vitro mucoadhesion test, which may contribute to intensifying the interaction with the mucosal membrane, promoting contact with the absorption site. Chitosan/HPMCP nanoparticles prolonged the drug release in a neutral environment; the release rates were significantly increased and sustained, governed by erosion, swelling, and diffusion mechanisms. Therefore, chitosan/HPMCP nanoparticles are potential systems to be explored for controlled release in mucosal routes of administration.

These results underscore the potential of this method for broader pharmaceutical applications and suggest that further investment in scaling up the polyelectrolyte complexation process is warranted. Additionally, post-processing steps, such as drying and resuspension, should be optimized, employing cryoprotectants like trehalose and nitrogen freezing to enhance uniformity.

Given the mucoadhesive properties of the NPs, nasal administration presents a promising route for direct delivery to the central nervous system via the olfactory pathway, potentially overcoming barriers like cellular efflux and the blood–brain barrier. Future work should focus on pharmacokinetic studies to further evaluate the transport and efficacy of nanoencapsulated MTX for glioblastoma treatment.

Several pharmaceutical excipients have been investigated concerning their ability to inhibit P-glycoprotein (P-gp)-mediated efflux in various chemotherapeutic agents. Surfactants and solubilizing excipients, such as caprylocaproyl polyoxyl-8 glycerides (Labrasol), polysorbates (Tween 20, Tween 80), polyoxyethylene esters of 12-hydroxystearic acid (Solutol HS-15), and poly(lactide)-tocopheryl polyethylene glycol succinate (TPGS), incorporate themselves between the lipophilic tails of bilayer membranes. This interaction modifies hydrogen bonding and ionic forces that maintain the membranes of the cells, improving the intestinal absorption of P-gp substrates via paracellular and transcellular pathways [46].

For glucan polysaccharides such as chitosan and hypromellose phthalate (HPMCP), the exact mechanism of P-gp efflux inhibition remains unclear. However, multiple studies have explored chitosan analogs and derivatives, demonstrating increased accumulation or absorption of P-gp substrates in vitro, in addition to enhancing the pharmacokinetic parameters. For instance, chitosan-4-thiobutylamidine (Ch-TBA) was evaluated in vivo in rats using rhodamine-123 (Rho-123) as a P-gp substrate. Enteric-coated tablets containing Ch-TBA increased the area under the plasma concentration-time curve of Rho-123 by 4.3-fold compared to the control [47].

Further studies synthesized three chitosan-4-thiobutylamidine (Ch-TBA) conjugates of varying molecular masses (Chito-9.4kDa-TBA, Chito-150kDa-TBA, and Chito-600kDa-TBA) and assessed their permeability effects on rat intestinal mucosa and Caco-2 monolayers using acyclovir as the P-gp substrate. Compared to the buffer alone, transport improvement was 1.3-fold with unmodified chitosan (0.5% *w*/*v*), 1.6-fold with Chito-150kDa-TBA (0.5% *w*/*v*), and 2.1-fold when combined with reduced glutathione (0.5% *w*/*v*) [48].

Yin et al. (2018) conducted in vitro studies on Caco-2 cells using various endocytosis inhibitors to investigate cellular uptake pathways [43]. They developed silybin-loaded N-octyl-O, and N-carboxymethyl chitosan micelles (OCC-SLB) to enhance silybin (SLB) oral absorption. Uptake studies showed that OCC-SLB micelles significantly enhanced the accumulation of SLB and rhodamine-123 in cells via clathrin- and caveolae-mediated endocytosis and P-gp efflux inhibition [43]. These findings highlight the potential of chitosan derivatives as functional excipients for P-gp efflux inhibition.

Expanding the scope to other biopolymers, Roy et al. (2014) [49] developed PEGylated carboxymethylcellulose conjugate nanoparticles (Cellax) loaded with docetaxel (DTX). Cellax therapy did not upregulate P-gp expression in MDA-MB-231 and EMT-6 breast tumor cells, in contrast to significant increases in P-gp mRNA and protein expression observed with native DTX. Additionally, Cellax demonstrated superior efficacy in a taxane-resistant breast tumor model, with a 6.5-fold lower *IC*_50_ against EMT6/AR1 cells compared to DTX and 90% tumor growth inhibition in vivo, while DTX showed no significant activity [49]. These results suggest that cellulose derivatives can also serve as functional excipients.

Regarding hypromellose derivatives, studies exploring their P-gp efflux inhibitory activity are limited. Our study introduced a novel approach by combining chitosan and HPMCP to enhance the uptake of nanoencapsulated methotrexate (MTX), a P-gp substrate, in glioblastoma cells. Cell viability assays comparing CCD 1059Sk fibroblasts and U251MG glioblastoma cells demonstrated the tumor-targeting specificity of MTX/QS/HPMCP-based nanoparticles. These nanoparticles showed superior efficacy if compared to free MTX and chitosan/TPP formulations.

Flow cytometry and confocal microscopy confirmed increased internalization and prolonged retention of MTX in glioblastoma cells when encapsulated in HPMCP/chitosan nanoparticles. This improvement may result from P-gp inhibition, enhancing MTX bioavailability and overcoming multidrug resistance. Such findings are particularly relevant for patients with rheumatoid arthritis or cancer, where P-gp overexpression limits MTX efficacy.

Compared to traditional P-gp inhibitors, excipient-based inhibitors exhibit minimal nonspecific pharmacological activity, reducing potential side effects [50]. Selecting functional excipients with low toxicity and P-gp inhibitory properties represents an innovative step in designing effective formulations for mucosal chemotherapy [46].

Based on prior art evidence already demonstrating the effect of chitosan in improving the absorption and targeting of chemotherapeutic agents, overcoming the P-gp efflux barrier, combined with the findings of this study showing an additive effect of HPMCP to enhance the MTX internalization in glioblastoma cells, this study can be a starting point for biomolecular and formulation scientists to explore HPMCP as a functional and safe excipient to improve cancer cell targeting of chemotherapy P-gp substrate drugs.

## 5. Conclusions

Our findings confirm the potential of MTX, HPMCP, and chitosan nanoparticles to selectively target and internalize within cancer cells, particularly glioblastoma cells, demonstrating effective control over MTX release, biocompatibility, mucoadhesion, and inhibition of P-glycoprotein (P-gp).

## Figures and Tables

**Table 1:** Proportions of chitosan (CS), hypromelose phthalate (HPMCP), and sodium tripolyphosphate (TPP) dispersions at 4 mg.mL^−1^, 2 mg.mL^−1^, 2 mg.mL^−1^, respectively, and final polymer concentrations tested for polyelectrolyte complexation to produce nanoparticle suspensions.

Samples	Polymers Proportions (*w*/*w*)		MTX (% *w*/*w*)	The Concentration of the Components (mg.mL^−1^)			pH
	QS	HPMCP		QS	HPMCP	MTX	SF
NPP29	3	1	-	1.82	0.60	-	5.6
NPPM23	3	1	0.05	1.82	0.60	0.0012	5.6
NPPM24	4	1	0.05	1.82	0.45	0.0011	5.6
NPPM25	6	1	0.05	1.82	0.30	0.0010	5.6
NPPM26	3	1	0.5	1.82	0.60	0.012	5.6
NPPM27	4	1	0.5	1.82	0.45	0.011	5.6
NPPM28	6	1	0.5	1.82	0.30	0.010	5.6
NPPM29	3	1	5	1.82	0.60	0.12	5.7
NPPM30	4	1	5	1.82	0.45	0.11	5.7
NPPM31	6	1	5	1.82	0.30	0.10	5.7
NPTM33	3	1	5	1.82	0.60	0.12	5.7
NPPM34	3	1	10	1.82	0.60	0.24	-
NPPM35	3	1	20	1.82	0.60	0.48	-

**Table 2:** Mean diameter, polydispersity index (PDI), zeta potential, and loading efficiency (*LE*%) of the nanoparticles. The values represent the mean and standard deviation of three replicates. NPP: chitosan and HPMCP nanoparticles, NPT: chitosan and TPP nanoparticles, M: loaded samples containing methotrexate at 5% *w*/*w*.

Samples	Mean Diameter (nm)	PDI	Zeta Potential (mV)	*LE*%
NPP29	456.80 ± 3.51	0.26 ± 0.01	21.40 ± 0.44	-
NPPM23	526.90 ± 14.36	0.27 ± 0.01	21.20 ± 1.13	36.17
NPPM24	580.43 ± 16.95	0.26 ± 0.01	24.80 ± 0.82	24.59
NPPM25	662.00 ± 19.03	0.26 ± 0.01	25.37 ± 1.59	26.30
NPPM26	456.00 ± 17.01 ^(a)^	0.26 ± 0.01	25.01 ± 1.03	6.60
NPPM27	558.17 ± 8.04	0.25 ± 0.02	24.20 ± 1.36	24.12
NPPM28	751.20 ± 25.15	0.34 ± 0.04	25.30 ± 1.04	38.94
NPPM29	452.60 ± 4.35 ^(a)^	0.25 ± 0.01	22.50 ± 0.41	91.72
NPPM30	600.57 ± 9.26	0.27 ± 0.01	22.67 ± 0.86	92.71
NPPM31	663.27 ± 3.59	0.25 ± 0.03	25.83 ± 0.21	89.60
NPTM33 ^1^	145.40 ± 1.90	0.22 ± 0.01	15.90 ± 1.01	15.37
NPPM34	*	*	*	*
NPPM35	*	*	*	*

^1^ NPTM33 was prepared under the same conditions as NPPM29 for comparison. * Parameters not evaluated due to the immediate drug precipitation. ^(a)^ Statistical significance difference between NPP-type nanoparticles (*p* > 0.05).

**Table 3:** Langmuir and Freundlich constants from Equations 1 and 2.

Samples	Freundlich (*)		Langmuir (**)		
	1/n	r^2^	a	b	r^2^
NPTM33	0.97	0.997	0.0001	0.1978	0.998
NPPM29	0.88	0.987	0.0011	0.1963	0.999

(*) $logQ_{e}=logk\frac{1}{n}xlogC_{e}$, (**) $\frac{1}{C_{e}}=a+bx\frac{1}{C_{e}}.$

**Table 4:** *IC*_50_ (μg.mL^−1^) calculated from the MTS assay for U251MG glioblastoma cell line. (NPP29) CS/HPMCP nanoparticles, (NPPM29) CS/HPMCP/MTX nanoparticles, (NPTM33) CS/TPP/MTX nanoparticles, and MTX (Methotrexate).

Samples	*IC*_50_ (μg.mL^−1^)	R^2^
NPP29	94.81	0.98
NPPM29	68.79	0.99
NPTM33	74.55	0.99
MTX	80.54	0.93

## Data Availability

The original contributions presented in the study are included in the article, further inquiries can be directed to the corresponding author/s.

## Supplementary Materials

The following supporting information can be downloaded at: https://www.mdpi.com/article/10.3390/pharmaceutics17020239/s1, Figure S1. FITIR of (a) chitosan, TPP and NPT33, (b) chitosan, HPMCP and NPP29 and (c) NPTM33, NPPM29 and MTX.
