# Supplementary material for: Uptake and Inhibition of P-Glycoprotein-Mediated Efflux Evaluation of Encapsulated Methotrexate Chitosan and Hypromellose Phthalate Nanoparticles for Potential Glioblastoma Treatment
Source: Pharmaceutics. 2025 Feb 12;17(2):239. doi: 10.3390/pharmaceutics17020239 (PMC11859166; doi:10.3390/pharmaceutics17020239)
Supplement: Supplementary file 1 [file pharmaceutics-17-00239-s001.zip › pharmaceutics-3241024-supplementary.pdf]

Supplementary material

Uptake and Inhibition of P-Glycoprotein-Mediated Efflux Evaluation of Encapsulated Methotrexate Chitosan and Hypromellose Phthalate Nanoparticles for Potential Glioblastoma Treatment

Valéria de Moura Leite Naves [1], Rafaela Franco Dias Bruzadelli [1], Marisa Ionta [3], Maria Palmira Daflon Gremião [4], Liliane Neves Pedreiro [5], Gislaine Ribeiro Pereira [6] and Flávia Chiva Carvalho [7,*]

3.1. FTIR Spectra Figures

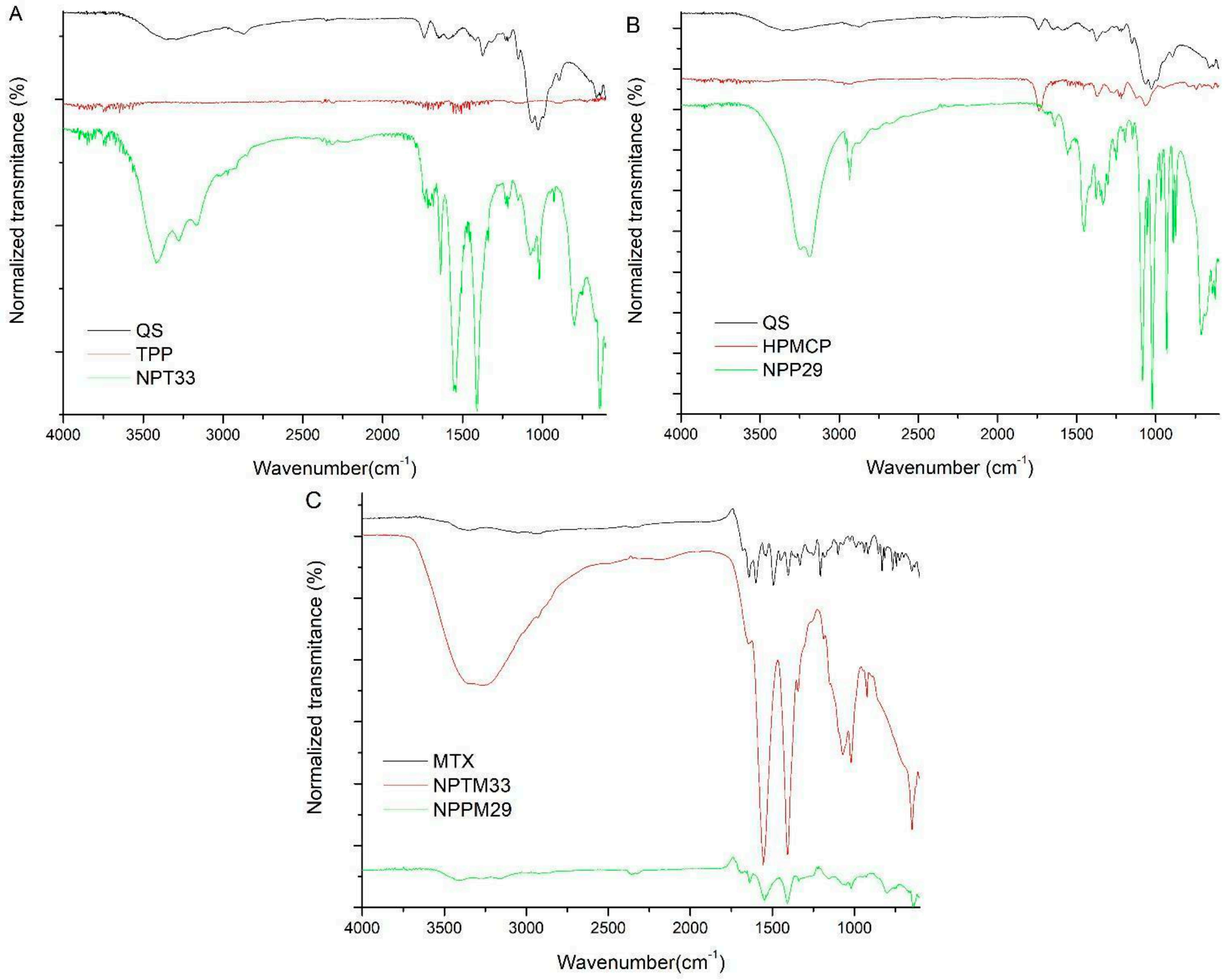

Figure S1. FITIR of (a) chitosan, TPP and NPT33, (b) chitosan, HPMCP and NPP29 and (c) NPTM33, NPPM29 and MTX.
